# Examining shift duration and sociodemographic influences on the well-being of healthcare professionals in the United Arab Emirates: a cross-sectional study

**DOI:** 10.3389/fpubh.2025.1517189

**Published:** 2025-02-25

**Authors:** Salaheddine Bendak, Iffat Elbarazi, Oumara Alajlouni, Sana O. Al-Rawi, Amal M. B. Abu Samra, Moien A. B. Khan

**Affiliations:** ^1^Faculty of Engineering, American University of Sharjah, Sharjah, United Arab Emirates; ^2^Faculty of Engineering, Haliç University, Istanbul, Türkiye; ^3^Institute of Public Health, College of Medicine and Health Sciences, United Arab Emirates University, Al-Ain, United Arab Emirates; ^4^Department of Medicine, University of Toronto, Toronto, ON, Canada; ^5^Department of Pediatrics, Al Qassimi Women and Children’s Hospital, Sharjah, United Arab Emirates; ^6^Internal Medicine, Abu Dhabi Health Services Co., Abu Dhabi, United Arab Emirates; ^7^Health and Wellness Research Group, Department of Family Medicine, College of Medicine and Health Sciences, United Arab Emirates University, Al-Ain, United Arab Emirates

**Keywords:** healthcare professionals, night shifts, work duration, sociodemographic characteristics, well-being

## Abstract

**Objective:**

Providing quality healthcare is an essential part of the healthcare system. The high workload and night work associated with healthcare providing may result in work-life imbalance among healthcare professionals (HCPs) and in degradation in the quality of care.

**Methods:**

This cross-sectional study aimed to assess the effects of sociodemographic characteristics and shift work on HCPs’ well-being in four large hospitals in the United Arab Emirates using a validated questionnaire.

**Results:**

Responses from 526 participants indicated that 79.2% of them were under the age of 40, 70.2% were females and 50.2% were single or divorced. Responses indicated that many HCPs reported engaging in unhealthy behaviors such as consuming energy drinks (37.3%), smoking (14.2%) and taking stimulant pills (5.7%) with males being more susceptible to these practices compared to females. Results also showed that participants perceived their overall health rate, on the average, to be moderate with one third of participants indicating to be overweight. Moreover, results showed that many participants reported having blood pressure issues (16%), diabetes (8.6%) and/or heart diseases (2.7%), with females more prone to these diseases than males. Another important finding was that most respondents reported sleeping significantly less than the recommended duration and taking too long time to fall asleep. Finally, results revealed that HCPs on 12-h shifts indicated having greater satisfaction but tend to experience more exhaustion levels and worsened health indicators than those on 8-h shifts.

**Conclusion:**

HCPs work has adverse effects on their well-being especially when combined with working at night. Healthcare implications of the results as well as recommendations to improve the well-being of HCPs based on the findings are given at the end.

## 1 Introduction and background

The provision of quality healthcare is of utmost importance for any healthcare professional (HCP). Over the years, there has been much debate regarding work-life imbalance of HCPs, which may, in turn, have adverse effects on the healthcare system. Understanding the emotional functioning of HCPs will help to improve the learning environment and, in turn, have a positive impact on the future of professionals within the healthcare system. HCPs are burdened with excessive workloads and shift (night) work, which contributes to a high rate of burnout, depression, and suicide among medical professionals (1). This can inevitably have a negative impact on the healthcare service quality.

Health and wellness consist of several components. These include a healthy diet, sleep duration and quality, spiritual wellbeing, physical activity, positive mood and emotional wellbeing (2). HCPs were identified as being at greater risk of absenteeism and poor well-being, eventually leading to lower retention and poor patient care outcomes (3, 4). This impact was heightened by the coronavirus disease pandemic (COVID-19) which led to a more negative impact on healthcare services and employees (5).

Besides this, shift work has become an integral part of many professions, with employees often working extended hours and irregular schedules to provide round-the-clock supply of goods and services (6, 7). Although this type of work arrangement is essential to maintain continuous and successful services in healthcare and many other fields, it can have significant adverse consequences on employees’ well-being and contribute to the growing issue of burnout (6, 8). It should be noted that professional burnout is a syndrome characterized by emotional exhaustion, feelings of cynicism or depersonalisation and decreased sense of personal accomplishment and is a recognized and concerning problem among physicians, with shift work being a potential exacerbating factor (9).

The demanding nature of HCPs’ work, coupled with long shifts, disrupts the body’s natural circadian rhythm and can lead to chronic fatigue, sleep disturbances and diminished neurocognitive functions (10, 11). As a result, they may experience a heightened risk of emotional and physical burnout and a sense of depersonalisation toward their patients (11). This not only affects the well-being of HCPs, but also has implications for patient care and the overall health system functioning, as also postulated by The Institution of Occupational Safety and Health (IOSH) (12).

Recent research has shed light on the various dimensions of how shift work impacts HCPs’ well-being (7, 13, 14). However, it underscored the need for a wide-ranging understanding of this issue to develop effective strategies for promoting physician well-being and ensuring optimal patient care. To mitigate the adverse effects of shift work on well-being of its employees, healthcare organizations are urged to implement supportive measures such as providing adequate rest periods, offering resources for stress management, and fostering a positive work environment that encourages work-life balance and professional growth (6, 12).

Many studies have identified various factors that are associated with anxiety, stress and poor well-being among HCPs. These factors include gender, marital status, number of children, nationality, service years, lack of social support, working hours and insufficient material and human resources (15–18).

Irregular schedules and prolonged shifts, which are common in many healthcare settings, have been exacerbated during the pandemic due to the huge demand on healthcare services and increased workload (19). In fact, their well-being during and following the COVID-19 pandemic became critical areas of concern that led to the demand for more concentrated wellness programs for HCPs and for more research in this area to better understand its dynamics especially in developing countries (20–22). The current cross-sectional study aimed to explore the multifaceted impact of sociodemographic characteristics and shift work on HCPs’ well-being. This is anticipated to improve planning of HCPs work schedules and training of HCPs as well as developing programs that help HCPs in coping with the high demands of their work.

## 2 Methodology

### 2.1 Study design and settings

An anonymous online cross-sectional survey was developed and administered for the purpose of the current study. The first part of the survey presented information on the study to allow potential volunteers to make an informed decision on participation. Then individuals were asked to indicate their consent to take part. Those who indicated not consenting to this study would then be automatically excluded from the survey. Those who indicated consenting to partipate in the study were directed to the questionionnaire for completion. The online English questionnaire portal was open for potential participants between 14 December 2021 and 28 March 2022 and took approximately 8–10 min to complete.

### 2.2 Inclusion and exclusion criteria

Participants eligible for inclusion in this study were:

- Healthcare professionals working in the United Arab Emirates, including physicians, nurses, midwives, and allied health workers.
- Aged 18 years or older.
- Currently employed in hospital settings with rotating or night shifts.
- Able to read and complete an English-language online survey.
- Willing to provide informed consent for participation.

Participants were excluded if they:

- Were not currently employed in a healthcare setting.
- Worked part-time or were in administrative roles unrelated to patient care.
- Were unable or unwilling to provide informed consent.
- Did not have proficiency in English, as the survey was administered exclusively in English.

### 2.3 Study population and sample size calculations

A sample size of 523 employees was calculated based on the sample size calculation criterion developed by Krejcie and Morgan (23). A conservative 95% confidence level and 5% margin of error were adopted where the population was estimated at 50,000 HCPs in the United Arab Emirates (UAE), the main setting of the study (24, 25). The inclusion criteria implemented in this study were that participants were expected to be HCPs working, working night shifts and willing to provide a consent. Participants were recruited through a snowball sampling method, where initial respondents were invited to distribute the survey link within their professional networks. Recruitment was conducted across four major hospitals in Abu Dhabi, Dubai, and Sharjah—the three largest emirates in the UAE—selected for their diverse workforce to ensure the inclusion of healthcare professionals from multiple disciplines. The questionnaire was exclusively distributed in English.

The data collection procedure involved sending emails directly to slightly more than 1,000 healthcare professionals. Additionally, the questionnaire link was circulated through WhatsApp groups within the same hospitals as a reminder. Eventually, a total of 589 subjects consented to participate with an estimated response rate of 54.9%. However, 63 surveys were not included in the analysis due to various reasons (mainly working part-time, currently not working or missing data). Consequently, a total of 526 surveys were considered in the current study.

### 2.4 Data collection

The current study employed a comprehensive set of validated questionnaires to assess various outcomes among participants. The methodology encompassed five distinct instruments where each instrument was carefully selected for its relevance and validated accuracy in capturing the data necessary for the current study’s objectives:

- Sociodemographic data: Gender, age, marital status, number of children at home <18 years of age, years of experience (26).
- Shiftwork data: Information on shift type and duration, years on night shifts (6, 27).
- Stimulants and substance abuse: Use of stimulants such as coffee, tea, energy drinks, and stimulant pills, smoking and alcohol consumption patterns (26).
- Health state: Participants’ health status was assessed based on overweight status, blood pressure, diabetes, heart disease, and a self-reported overall health rating on a scale from 0 to 100, where 0 indicated “poor health” and 100 indicated “excellent health.” This single-item measure is widely used in population health research as a subjective indicator of well-being and has been shown to correlate with objective health measures, including chronic disease burden and healthcare utilization (28).
- Occupational well-being: Occupational well-being was assessed using selected items from the Professional Fulfillment Index (PFI) (2) a validated measure designed to evaluate burnout and professional fulfillment among healthcare professionals. The PFI includes:Four items assessing work exhaustion (‘I feel emotionally drained from my work’), Four items evaluating interpersonal disengagement (‘I have become more callous toward people since I took this job’), and Six items measuring professional fulfillment (‘I find meaning in my work’). Each item was rated on a 5-point Likert scale ranging from 0 (‘not at all’) to 4 (‘extremely’) for burnout items, and from 0 (‘not at all true’) to 4 (‘completely true’) for professional fulfillment items. Scores were summed within each subscale, yielding possible scores from 0 to 40 for burnout and from 0 to 24 for professional fulfillment. Higher scores on burnout subscales indicate greater work-related distress, whereas higher professional fulfillment scores indicate greater job satisfaction and meaningfulness.

### 2.5 Study outcome

The dependent variables are HCPs’ well-being, stimulants use, substance abuse and medical history, while the independent variables included sociodemographic factors, shift work and shift duration.

### 2.6 Statistical analysis

In this study, all collected data had undergone a numerical coding process. For categorical data, percentages and frequencies were used to provide an overview of the distribution of responses. For numerical data, the mean and standard deviation were calculated to provide a central measure and an indication of data’s spread. Then, *t*-test, non-parametric (namely Kruskal-Wallis and Mann-Wittney *U*) tests and cross tabulation were used to analyze the data. The level of statistical significance was set at 0.05, meaning that any findings with *p*-values less than 0.05 would be considered statistically significant. Statistical analysis was carried out using SPSS version 27.

### 2.7 Ethical approval

This study adhered to the ethical guidelines outlined in the Helsinki Declaration (29). It was approved by the Ethics Committee for Social Sciences at the United Arab Emirates University (ERS_2021_8423). Informed consent was secured from all participants prior to the start of the research, ensuring compliance with ethics procedures.

## 3 Results

As indicated earlier, out of the 549 HCPs who responded to the questionnaire, 526 were considered for the current study. Out of those 526, 369 (70.2%) were female and 264 (50.2%) indicated that they were single or divorced. Moreover, 114 (21.7%) of respondents were 21–25 years of age, 133 (25.3%) were between 26 and 30, 119 (22.6%) between 31 and 35, 51 (9.7%) between 36 and 40, 39 (7.4%) between 41 and 45, 33 (6.3%) between 46 and 50, 31 (5.9%) between 51 and 55, and 6 (1.2%) were 56 years of age or older. For the number of children under the age of 18, 181 (34.4%) said they had no children, 110 (20.9%) indicated that they have one child, 119 (22.6%) two children, 48 (9.1%) three children, 24 (4.6%) four children, 39 (7.4%) five children, and 3 (0.6%) six children.

For type of professional practice, responses indicated that 341 (64.8%) of participants were medical doctors, 159 (30.2%) were nurses, 21 (4%) were midwives and 5 (1%) were others. For continuous recent work experience on night shifts, the average was 1.77 years (stdev = 1). Finally, for night shift duration, 256 (48.7%) respondents indicated working 8-h night shifts and 270 (43.7%) indicated working 12-h night shifts. All of the respondents indicated working rotating shift schedules with none working on permanent night shifts.

The average weight of participants was 73.1 kg with a standard deviation of 17.9 and the average height was 166.8 cm with a standard deviation of 9.9. Regarding body image, 66.2% of participants reported not feeling overweight, while 33.3% do. Hypertension was present in 16% of participants, while the prevalence of diabetes and heart diseases were 8.6 and 2.7%, respectively. The overall health rate was 71.3%, with a standard deviation of 21.3.

**Figure 1:**
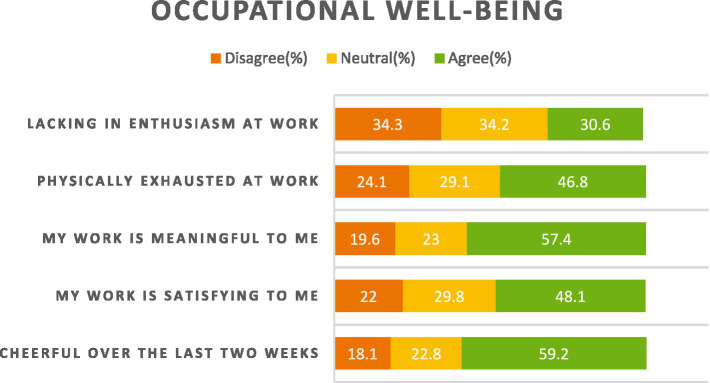
Occupational well-being survey responses.

Questionnaire results related to behavior and consumption are summarized in Table 1 and responses to well-being questions are illustrated in Figure 1. To simplify both Table 1 and Figure 1, strongly disagree and disagree were summed up into one category (disagree) and agree and strongly disagree were summed up into one category (agree).

**Table 1:** Summary of responses to questions related to behavior and consumption.

Behavior/Consumption category	Level 1	Level 2	Level 3
Coffee	No 28.1%	1–2 cups 35%	>3 cups 36.9%
Tea	No 45.8%	1–2 cups 23.6%	>3 cups 30.6%
Energy drinks	No 62.7%	yes 37.3%	
Stimulant pills	No 94.3%	yes 5.7%	
Cigarettes per day	No 85.4%	<5 cig 3.8%	>6 cig 10.8%
Alcohol per week	No 87.3%	1–2 drinks 9.7%	>2 drinks 3%

Questionnaire results indicate that 37.3% of participants consume energy drinks, 5.7% use stimulant pills, 14.6% smoke cigarettes and 10.7% consume alcohol. Moreover, 33.3% reported perceiving themselves to be overweight, 16% indicated having blood pressure problems, 8.6% indicated having diabetes and 2.7% indicated having heart diseases. Interestingly, respondents indicated perceiving, on the average, their health rate to be 71.3% which is considered a, relatively speaking, low average. Finally, 59.2% indicted feeling cheerful at work, 48.1% indicated being satisfied at work, 57.4% indicated their work to be meaningful to them, 46.8% indicated being physically exhausted at work and 30.6% indicated lacking enthusiasm at work.

### 3.1 Gender differences

Statistically significant differences in response factors due to gender are summarized in Table 2. As can be seen in that table, male HCPs interestingly reported significantly consuming more coffee, energy drinks, stimulant pills, cigarettes and alcohol while, at the same time, rating their overall health to be worse than female participants. Nevertheless, female participants reported to have significantly higher occurrences of blood pressure and diabetes than male participants.

**Table 2:** Statistically significant differences in output factors due to gender.

Independent factor		Level 1	Level 2	Level 3	Level 4	*p*-value
Coffee consumption	Males	None 23.6%	1–2 cups 29.9%	≥3 cups 46.5%		*p* = 0.008
	Females	None 30.1%	1–2 cups 37.1%	≥3 cups 32.8%		
Energy drinks consumption	Males	No 47.1%	yes 52.9%			*p* < 0.001
	Females	No 69.4%	yes 30.6%			
Using stimulant pills	Males	No 89.9%	yes 10.1%			*p* = 0.004
	Females	No 96.2%	yes 3.8%			
Smoking cigarettes	Males	No 66.2.3%	<5 cig 5.4%	5–10 cig 19.1%	>11 cig 8.9%	*p* < 0.001
	Females	No 93.5%	<5 cigarettes 3%	5–10 cigarettes 2.4%	>11 cig 1.1%	
Alcohol consumption	Males	No 76.4%	1–3 drinks 19.1%	4–6 drinks 2.5%	>7 drinks 1.9%	*p* = 0.001
	Females	No 91.1%	1–3 drinks 5.7%	4–6 drinks 1.4%	>7 drinks 1.1%	
Having blood pressure	Males	No 89.8%	yes 10.2%			*p* = 0.009
	Females	No 81.6%	yes 18.4%			
Having diabetes	Males	No 94.9%	yes 5.1%			*p* = 0.031
	Females	No 90%	yes 10%			
Health rate	Males	Ave 67.7% SD (21.4)				*p* = 0.038
	Females	Ave 72.3 SD (21.2)				

### 3.2 Marital status

Statistically significant differences in response factors due to marital status are summarized in Table 3. As can be seen in that table, percentage of married respondents consuming more tea and alcohol is significantly greater than percentage of single respondents. Also, percentage of married HCPs reported perceiving their work to be meaningful is significantly greater than that of single HCPs.

**Table 3:** Statistically significant differences in output factors due to marital status.

Independent factor		Level 1	Level 2	Level 3	Level 4	Level 5	*p*-value
Tea consumption	Single	No 48.5%	1–2 cups 25%	≥3 cups 26.5%			*p* = 0.035
	Married	No 43.1%	1–2 cups 22.1%	≥3 cups 34.7%			
Alcohol consumption	Single	No 90.2%	1–3 drinks 7.2%	4–6 drinks 1.9%	>7 drinks 0.8%		*p* = 0.04
	Married	No 84.4%	1–3 drinks 12.2%	4–6 drinks 1.5%	>7 drinks 1.9%		
My work is meaningful to me	Single	Strongly disagree 3%	Disagree 15.9%	Neutral 28.8%	Agree 34.5%	Strongly agree 17.8%	*p* = 0.023
	Married	Strongly disagree 5.7%	Disagree 14.5%	Neutral 17.2%	Agree 31.3%	Strongly agree 31.3%	

### 3.3 Age

There was significant difference in tea intake due to age of respondents (*p* < 0.001). Results showed clearly that younger HCPs drink less tea than their older counterparts. This was seen with the uniform drop of percentage of tea non-drinkers from 65.8% for 20–25 age group to 16.1% for 51–55 age group.

There was also a significant perception difference in being overweight among respondents. The ratio of respondents perceiving themselves to be overweight was fluctuating between 60 and 68% till the age of 50 to jump then to 77% for 51–55 age group and then to 80% for 56–60 age group (*p* < 0.001). This might be attributed to the slowing down of metabolism and menopause after the age of 50.

### 3.4 Number of children

Number of children did not have any meaningful significant effect on any of the output variables.

### 3.5 Shift duration

Statistically significant differences in response factors due to shift duration are summarized in Table 4. As can be seen in that table, HCPs on 8-h shifts tend to consume more tea and energy drinks, use stimulant pills and report to be overweight more than those on 12-h shifts. Also, HCPs on 8-h shifts reported significantly lower work satisfaction rate and perceived their work to be less meaningful than those on 12-h shifts. Nevertheless, HCPs on 8-ht shifts reported to be significantly less exhausted at work than those on 12-h shifts.

**Table 4:** Statistically significant differences in output factors due to night shift length.

Independent factor	Shift duration	Level 1	Level 2	Level 3	Level 4	Level 5	*p*-value
Tea consumption	8-h	None 40.2%	1–2 cups 18.9%	≥3 cups 41.8%			*p* = 0.000
	12-h	None 51.1%	1–2 cups 28.9%	≥3 cups 20%			
Energy drinks consumption	8-h	No 77.4%	Yes 42.2%				*p* = 0.014
	12-h	No 67.8%	Yes 32.2%				
Using stimulant pills	8-h	No 91%	Yes 9%				*p* = 0.001
	12-h	No 97.4%	Yes 2.6%				
Being overweight	8-h	No 62.5%	Yes 37.5%				*p* = 0.009
	12-h	No 69.6%	Yes 30.4%				
My work is satisfying to me	8-h	Strongly disagree 3.1%	Disagree 23%	Neutral 35.2%	Agree 28.9%	Strongly agree 9.8%	*p* = 0.001
	12-h	Strongly disagree 6.7%	Disagree 11.5%	Neutral 24.8%	Agree 38.9%	Strongly agree 11.1%	
My work is meaningful to me	8-h	Strongly disagree 2%	Disagree 18%	Neutral 34.4%	Agree 33.2%	Strongly agree 12.5%	*p* = 0.000
	12-h	Strongly disagree 6.7%	Disagree 12.6%	Neutral 12.2%	Agree 32.6%	Strongly agree 35.9%	
I feel physically exhausted at work	8-h	Strongly disagree 5.5%	Disagree 22.3%	Neutral 31.6%	Agree 24.2%	Strongly agree 16.4%	*p* = 0.014
	12-h	Strongly disagree 5.9%	Disagree 14.8%	Neutral 26.7%	Agree 30%	Strongly agree 22.6%	

### 3.6 Years on night shift

There were no significant differences in output variables due to years on night shift. This might be attributed to the short duration spent on night shifts by healthcare professionals participating in this study (ranging between 0 and 3 years).

## 4 Discussion

This study explored demographics and shift work influence on a sample of healthcare professionals’ well-being in the UAE HCPs. Study results indicate that some HCPs, like other professionals, engage in unhealthy practices like consuming stimulant pills and smoking. Although most of participating HCPs are young, many perceived their health not to be that good on average (71.3%). Results also show that 16% of participants have blood pressure issues, 8.6% have diabetes and 2.7% have heart diseases, which might be considered high given the young age of most participants. Besides this, one third of participants feel they are overweight with this feeling significantly increasing for those above the age of 50 when metabolism slows down and people tend to gain weight more easily as reported before (30).

As anticipated, male healthcare professionals (HCPs) revealed higher consumption of coffee, energy drinks, stimulant pills, cigarettes and alcohol compared to their female counterparts, aligning with the consumption norms and patterns observed for these substances (31). However, female participants reporting to have higher occurrences of blood pressure and diabetes than male participants does not match the general trend in the society and is a topic which should be investigated further in future studies (32, 33). Further research is also needed to explore if gender-based awareness programs are needed in terms of stress management and lifestyle.

As for marital status, the percentage of married respondents consuming more tea and alcohol was found to be greater than that of single respondents contrary to a study conducted among females in Saudi Arabia (34). Also, the percentage of married participants perceiving their work to be meaningful was found to be greater than that of single participants. This latter result might be attributed to the psychological dynamics of having a family which brings with it a sense of responsibility and the need to work (35).

Moreover, many participants reported having blood pressure issues, diabetes and/or heart diseases, with females more prone to these diseases than males, although participants were mainly young. Besides this, most respondents reported sleeping less than recommended duration and taking too long time to sleep. This raises concerns on the long-term health and well-being of HCPs and warrants further research in this area.

The above results revealed that HCPs on 8-h shifts tend to consume more tea and energy drinks, use stimulant pills and report to be overweight more than those on 12-h shifts. Alongside these results, HCPs on 8-h shifts reported lower work satisfaction rate and perceived their work to be less meaningful than those on 12-h shifts. In parallel, HCPs on 8-ht shifts reported to be significantly less exhausted at work than those on 12-h shifts. These results indicate that HCPs are generally more satisfied with 12-h shifts and found their work to be more meaningful than those 8-h shifts which was also reported in the literature. Reasons might include less trips to and from work, more time with family on days off, greater opportunity of taking another job, etc. (6). At the same time, participants also reported that 12-h shifts are significantly more exhausting than 8-h shifts. This higher exhaustion levels may be attributed to potential shorter sleep duration between consecutive shifts, sleep deprivation, higher stress levels, accumulated fatigue during the extra 4 hours at work, disrupted physical activity and dietary habits due to longer shifts, and possibly other factors. It is recommended, thus, that long term effects of this potential high exhaustion attributed to 12-h shifts should be further investigated in future studies (6, 35, 36).

Findings of the current study suggest a need for targeted health interventions for shift HCPs, focusing on lifestyle modifications, sleep health and stress management. These interventions should also address the high use of stimulants and associated risks. The study also highlights the importance of routine screenings of health and sleeping habits of shift workers among HCPs. Clinicians should be aware of the unique challenges faced by this group and provide resources for managing work-related stress and sleep debt. Moreover, this study underlines the necessity of integrating occupational health into medical and nursing education.

The present study had an obvious limitation in that the causal relationship between sociodemographic factors and shiftwork on HCPs’ well-being could not be inferred from this cross-sectional study. Future research should focus on longitudinal studies to assess the long-term impacts of shift work on health. A major strength in the current study was the use of validated instruments for data collection, thus enhancing the reliability of the findings. However, the cross-sectional design had a potential for response bias. In addition, limited generalisability was possible due to the specific demographic and geographic location of the participants.

## 5 Conclusion

Identifying factors that affect the well-being of healthcare professionals (HCPs) is of utmost importance for the development of effective strategies to improve their working conditions and the quality of patient care. Results of the current study revealed that many healthcare professionals (HCPs) have unhealthy practices, like smoking and consuming energy drinks, with males being more prone to these practices than female HCPs. Participants also indicated perceiving their overall health rate, on the average, to be moderate with one third of participants indicating to be overweight. These findings are exacerbated further when HCPs were involved with shiftwork. These findings emphasize the importance of paying more attention to the health and well-being of HCPs through improving their work conditions and giving them adequate psychological support.

## Data Availability

The raw data supporting the conclusions of this article will be made available by the authors, without undue reservation.

## References

[1] Sharifi M Asadi-Pooya AA Mousavi-Roknabadi RS. Burnout among healthcare providers of COVID-19; a systematic review of epidemiology and recommendations. Arch Acad Emerg Med. (2020) 9:e7. doi: 10.22037/aaem.v9i1.1004, PMID: 33490964 PMC7812159

[2] Trockel M Bohman B Lesure E Hamidi MS Welle D Roberts L. A brief instrument to assess both burnout and professional fulfillment in physicians: reliability and validity, including correlation with self-reported medical errors, in a sample of resident and practicing physicians. Acad Psychiatry. (2018) 42:11–24. doi: 10.1007/s40596-017-0849-3, PMID: 29196982 PMC5794850

[3] Shanafelt TD Boone S Tan L Dyrbye LN Sotile W Satele D. Burnout and satisfaction with work-life balance among US physicians relative to the general US population. Arch Intern Med. (2012) 172:1377–85. doi: 10.1001/archinternmed.2012.3199, PMID: 22911330

[4] Ferri P Guadi M Marcheselli L Balduzzi S Magnani D Di Lorenzo R. The impact of shift work on the psychological and physical health of nurses in a general hospital: a comparison between rotating night shifts and day shifts. Risk Manag Healthc Policy. (2016) 9:203–11. doi: 10.2147/RMHP.S115326, PMID: 27695372 PMC5028173

[5] Alkhamees AA Aljohani MS Kalani S Ali AM Almatham F Alwabili A. Physician’s burnout during the COVID-19 pandemic: a systematic review and Meta-analysis. Int J Environ Res Public Health. (2023) 20:4598. doi: 10.3390/ijerph20054598, PMID: 36901612 PMC10001574

[6] Bendak S. 12-h workdays: current knowledge and future directions. Work Stress. (2003) 17:321–36. doi: 10.1080/02678370310001643478

[7] Costa G. Shift work and health: current problems and preventive actions. Saf Health Work. (2010) 1:112–23. doi: 10.5491/SHAW.2010.1.2.112, PMID: 22953171 PMC3430894

[8] De Hert S. Burnout in healthcare workers: prevalence, impact and preventative strategies. Local Reg Anesth. (2020) 13:171–83. doi: 10.2147/LRA.S240564, PMID: 33149664 PMC7604257

[9] Park S Porter M Park K Bielick L Rooks BJ Mainous AG III. What are the characteristics of fourth-year medical students with higher levels of resilience?. PRiMER: peer-review reports in medical education. Research. (2019) 3:381. doi: 10.22454/PRiMER.2019.150381, PMID: 32537593 PMC7205096

[10] Caruso CC. Negative impacts of shiftwork and long work hours. Rehabil Nurs. (2014) 39:16–25. doi: 10.1002/rnj.107, PMID: 23780784 PMC4629843

[11] Maslach C Leiter MP. Understanding the burnout experience: recent research and its implications for psychiatry. World Psychiatry. (2016) 15:103–11. doi: 10.1002/wps.20311, PMID: 27265691 PMC4911781

[12] The Institution of Occupational Safety and Health (2015). The effects of shift work on health. Available at: https://iosh.com/media/12200/iosh-research-effects-of-shift-work-on-health-full-report.pdf (Accessed February 17, 2023).

[13] Wisetborisut A Angkurawaranon C Jiraporncharoen W Uaphanthasath R Wiwatanadate P. Shift work and burnout among health care workers. Occup Med. (2014) 64:279–86. doi: 10.1093/occmed/kqu009 24550196

[14] Klinefelter Z Hirsh EL Britt TW George CL Sulzbach M Fowler LA. Shift happens: emergency physician perspectives on fatigue and shift work. Clocks Sleep. (2023) 5:234–48. doi: 10.3390/clockssleep5020019, PMID: 37092431 PMC10123702

[15] Aljabri D Alshatti F Alumran A Al-Rayes S Alsalman D Althumairi A. Sociodemographic and occupational factors associated with burnout: a study among frontline healthcare workers during the COVID-19 pandemic. Front Public Health. (2022) 10:854687. doi: 10.3389/fpubh.2022.854687, PMID: 35356019 PMC8959574

[16] Althumairi A Ayed AlOtaibi NM Alumran A. Alrayes S and Owaidah a factors associated with anxiety symptoms among medical laboratory professionals in Khobar: single institution study. Front Public Health. (2022) 10:917619. doi: 10.3389/fpubh.2022.917619, PMID: 36159270 PMC9500507

[17] Elbarazi I Loney T Yousef S Elias A. Prevalence of and factors associated with burnout among health care professionals in Arab countries: a systematic review. BMC Health Serv Res. (2017) 17:491. doi: 10.1186/s12913-017-2319-8, PMID: 28716142 PMC5513024

[18] Estryn-Behar M van der Heijden BI Fry C Hasselhorn HM. Longitudinal analysis of personal and work-related factors associated with turnover among nurses. Nurs Res. (2010) 59:166–77. doi: 10.1097/NNR.0b013e3181dbb29f, PMID: 20421841

[19] Gupta N Dhamija S Patil J Chaudhari B. Impact of COVID-19 pandemic on healthcare workers. Ind Psychiatry J. (2021) 30:S282–4. doi: 10.4103/0972-6748.328830, PMID: 34908710 PMC8611576

[20] Alrawashdeh HM Al-Tammemi AB Alzawahreh MK Al-Tamimi A Elkholy M Al Sarireh F. Occupational burnout and job satisfaction among physicians in times of COVID-19 crisis: a convergent parallel mixed-method study. BMC Public Health. (2021) 21:811. doi: 10.1186/s12889-021-10897-4, PMID: 33906619 PMC8079229

[21] Malik H Annabi CA. The impact of mindfulness practice on physician burnout: a scoping review. Front Psychol. (2022) 13:956651. doi: 10.3389/fpsyg.2022.956651, PMID: 36204751 PMC9530040

[22] El Dabbah NA Elhadi YAM. High levels of burnout among health professionals treating COVID-19 patients in two Nile basin countries with limited resources. Sci Rep. (2023) 13:6455. doi: 10.1038/s41598-023-33399-2, PMID: 37081113 PMC10116483

[23] Krejcie R Morgan D. Determining sample size for research activities. Educ Psychol Meas. (1970) 30:607–10. doi: 10.1177/001316447003000308

[24] Emirates News Agency. (2022). Number of medical personnel in UAE increased by 140% over 10 years: FCSC. Available at: https://wam.ae/en/details/1395303015848 (Accessed February 17, 2023).

[25] Chemali Z Ezzeddine FL Gelaye B Dossett ML Salameh J Bizri M. Burnout among healthcare providers in the complex environment of the Middle East: a systematic review. BMC Public Health. (2019) 19:1337. doi: 10.1186/s12889-019-7713-1, PMID: 31640650 PMC6805482

[26] Abdulle A Alnaeemi A Aljunaibi A Al Ali A Al Saedi K Al Zaabi E. The UAE healthy future study: a pilot for a prospective cohort study of 20,000 United Arab Emirates nationals. BMC Public Health. (2018) 18:101–9. doi: 10.1186/s12889-017-5012-2, PMID: 29304844 PMC5755402

[27] Poissonnet CM Véron M. Health effects of work schedules in healthcare professions. J Clin Nurs. (2000) 9:13–23. doi: 10.1046/j.1365-2702.2000.00321.x, PMID: 11022488

[28] Hall T Krahn GL Horner-Johnson W Lamb G. Examining functional content in widely used health-related quality of life scales. Rehabil Psychol. (2011) 56:94–9. doi: 10.1037/a0023054, PMID: 21574727

[29] World Medical Association. World medical association declaration of Helsinki: ethical principles for medical research involving human subjects. J Am Coll Dent. (2014) 81:14–8. 25951678

[30] Fan W. Epidemiology in diabetes mellitus and cardiovascular disease. Cardiovasc Endocrinol. (2017) 6:8–16. doi: 10.1097/XCE.0000000000000116, PMID: 31646113 PMC6768526

[31] Roemer A Stockwell T Zhao J Chow C Vallance K Cherpitel C. Gender differences in the consumption of alcohol mixed with caffeine and risk of injury. Drug Alcohol Rev. (2019) 38:750–7. doi: 10.1111/dar.12997, PMID: 31599075 PMC6907685

[32] Wang Z Yang T Fu H. Prevalence of diabetes and hypertension and their interaction effects on cardio-cerebrovascular diseases: a cross-sectional study. BMC Public Health. (2021) 21:1224. doi: 10.1186/s12889-021-11122-y, PMID: 34172039 PMC8229421

[33] Ciarambino T Crispino P Leto G Mastrolorenzo E Para O Giordano M. Influence of gender in diabetes mellitus and its complication. Int J Mol Sci. (2022) 23:8850. doi: 10.3390/ijms23168850, PMID: 36012115 PMC9408508

[34] Alfawaz HA Khan N Yakout SM Khattak MNK Alsaikhan AA Almousa AA. Prevalence, predictors, and awareness of coffee consumption and its trend among Saudi female students. Int J Environ Res Public Health. (2020) 17:7020. doi: 10.3390/ijerph17197020, PMID: 32992846 PMC7579070

[35] Hansen AB Stayner L Hansen J Andersen ZJ. Night shift work and incidence of diabetes in the Danish nurse cohort. Occup Environ Med. (2016) 73:262–8. doi: 10.1136/oemed-2015-103342, PMID: 26889020

[36] Pallesen S Bjorvatn B Magerøy N Saksvik IB Waage S Moen BE. Measures to counteract the negative effects of night work. Scand J Work Environ Health. (2010) 36:109–20. doi: 10.5271/sjweh.2886 20011984

